# Preoperative Serum Cystatin C as an Independent Prognostic Factor for Survival in Patients with Renal Cell Carcinoma

**DOI:** 10.7150/jca.97711

**Published:** 2024-09-23

**Authors:** Hui Ma, Peipei Wang, Zhao Hou, Huiyu Zhou, Dingyang Lv, Fan Cui, Weibing Shuang

**Affiliations:** 1 School of Public Health, Shanxi Medical University, Taiyuan 030001, China.; 2 Grand Hospital of Shuozhou, Shuozhou 036000, China.; 3 Academy of Medical Sciences, Shanxi Medical University, Taiyuan 030001, China.; 4 First Clinical Medical College, Shanxi Medical University, Taiyuan 030001, China.; 5 Assisted Reproductive Center, Taiyuan Hospital of Peking University First Hospital, Taiyuan 030032, Shanxi, China.; 6 Department of Urology, The First Hospital of Shanxi Medical University, No. 85, JieFang South Road, Yingze District, Taiyuan 030001, China.

**Keywords:** Renal cell carcinoma, Cystatin C, Survival, Prognosis

## Abstract

**Purpose:** This study aims to evaluate the prognostic significance of preoperative serum cystatin C (Cys-C) in patients with renal cell carcinoma (RCC).

**Methods:** We analyzed clinicopathological data and follow-up information of 624 RCC patients who underwent partial or radical nephrectomy at our institution. The optimal cutoff value of Cys-C was determined using X-tile software. Survival outcomes, including overall survival (OS) and cancer-specific survival (CSS), were evaluated using the Kaplan-Meier method and log-rank test. To avoid overfitting and collinearity, we used LASSO-based multivariable Cox regression analysis to identify independent predictors of OS and CSS. The predictive accuracy of the established model, including preoperative serum Cys-C, was evaluated using the time-dependent receiver operating characteristic (ROC) curves and the area under the curve (AUC).

**Results:** The median follow-up period was 40 months. The optimal cutoff value of preoperative serum Cys-C levels was 0.95 mg/L. Compared with the low Cys-C group, patients in the high Cys-C group had significantly shorter OS and CSS. Multivariable Cox regression analysis indicated that elevated preoperative serum Cys-C level was an independent adverse predictor for RCC patients post-nephrectomy. After adjusting for all covariates, high preoperative serum Cys-C level was associated with worse OS (hazard ratio [HR]: 2.254; 95% confidence interval [CI]: 1.144, 4.439; *P* = 0.019) and CSS (HR: 3.621; 95% CI: 1.386, 9.456; *P* = 0.009). Time-dependent ROC analysis demonstrated that our model, including preoperative serum Cys-C, performed well in predicting accuracy of survival.

**Conclusions:** Preoperative serum Cys-C level is an effective prognostic indicator for OS and CSS in RCC patients undergoing nephrectomy.

## Introduction

Renal cell carcinoma (RCC), one of the most lethal urogenital malignancies, originates from renal tubular epithelial cells and accounts for 80-90% of all renal neoplasms 1. According to Global Cancer Statistics 2020, RCC occurs mostly in European and North American populations 2. However, the incidence of RCC in Asia is projected to rise as nation's transition to a Western lifestyle 3. The most important cause of such an increase is the advancements in medical imaging, such as CT or MRI, and easily accessible abdominal imaging facilities 4. Although the diagnosis and treatment of RCC have made unquestionable progress, the 5-year survival rate remains unsatisfactory 5, 6. To evaluate postoperative risks and optimize individualized therapy, various traditional prediction factors, including Fuhrman grade, TNM classification, and tumor size, have been closely associated with patient survival following RCC surgery. However, these parameters are not perfectly accurate when used alone 7. Therefore, it is necessary to combine more accessible laboratory parameters as prognostic indicators to better assess outcomes in RCC patients.

Cystatin C (Cys-C), a potent inhibitor of lysosomal cysteine proteinases, is produced by all nucleated cells and released into the bloodstream 8. The main catabolic site of Cys-C is the kidney, where it is almost completely freely filtered by the glomerulus and enzymatically degraded after complete reabsorption in the proximal tubule 8. Since these properties meet most criteria for being an ideal marker of glomerular filtration rate, Cys-C has long been considered as an indicator to evaluate renal function 9, 10. Besides its proteinase inhibiting activity, Cys-C also regulates other important biological functions, including cell proliferation 11, cell differentiation 12, cell migration 13, and immune modulation 14. Previous studies have demonstrated a significant association between elevated serum Cys-C and poor survival outcomes in patients with various solid tumors, such as nasopharyngeal carcinoma 15, lung cancer 16, gastrointestinal tumors 17, breast cancer 18 or with hematological malignancies 19, 20. However, few studies have investigated its prognostic value in RCC.

Hence, this study aims to assess the prognostic impact of preoperative serum Cys-C on RCC patients undergoing nephrectomy.

## Patients and methods

### Patients

We retrospectively collected clinicopathological data from 1,067 RCC patients who underwent partial or radical nephrectomy at the Department of Urology, First Hospital of Shanxi Medical University (Taiyuan, China) between 2013 and 2021.The exclusion criteria were as follows: (1) incomplete clinical and pathological data (n=357); (2) presentation with other malignant diseases (n=32); (3) receiving preoperative anticancer therapy (n=14); (4) perioperative death (n=2); (5) loss to follow-up (n=38). Finally, a total of 624 eligible patients were included in our study. This study was reviewed and approved by the Ethics Committee of the First Hospital of Shanxi Medical University, and all patients enrolled had provided written informed consent.

### Data collection and follow-up

All relevant clinicopathological data were collected from the electronic medical records, including gender, age, body mass index (BMI), smoking, diabetes, hypertension, cardiovascular disease, tumor laterality, tumor size, tumor subtype, T stage, N stage, Fuhrman grade, type of surgery, surgical approach, and preoperative urea, creatinine (CRE), and uric acid (UA) levels. Preoperative serum Cys-C levels were measured from routine blood tests one day after patient admission. Its optimal cutoff point was determined using X-tile 3.6.1(Yale University, USA), and then patients were classified into low and high Cys-C groups.

Follow-up was conducted via outpatient visits or telephone calls post-treatment. Follow-up intervals were every 3 months during the first 3 years, every 6 months during years 4 and 5, and annually thereafter. The main endpoint was overall survival (OS), defined as the time (months) from the date of surgery to death or last follow-up, which was September 30, 2022. The secondary endpoint was cancer-specific survival (CSS), defined as the time (months) from the date of surgery to cancer-related death or last follow-up.

### Statistical analysis

Continuous variables were presented as mean ± standard deviation and analyzed by the Student's t-test. Categorical variables were expressed as frequency (percentage) and analyzed by the Chi-squared test. The optimal cutoff point of Cys-C levels was determined using X-tile software. The impact of Cys-C on survival outcomes was evaluated using the Kaplan-Meier curves and log rank test. Significant variables in the LASSO regression analysis were included in multivariate Cox regression model to identify independent predictors of survival. In further analysis, Cox proportional hazards regression models were used to evaluate the association between preoperative serum Cys-C and OS or CSS in RCC patients. We set up models as follows: covariates unadjusted model; model Ⅰ adjusted for age, gender, and BMI; model Ⅱ additionally adjusted for laterality, smoking history, diabetes, hypertension, and cardiovascular disease based on model Ⅰ; model Ⅲ adjusted for all covariates. The time-dependent receiver operating characteristic (ROC) curves and the area under the curve (AUC) were used to assess the predictive value of the established model. All data analyses were performed using SPSS 25.0 (IBM Corp., Armonk, NY, USA) and R version 4.3.2. Differences were considered statistically significant when *P* < 0.05.

## Results

### The characteristics of patients

Among the 624 patients, 408 (65.4%) were male and 216 (34.6%) were female. The mean age was 57.49 years (range: 27-86 years). The optimal cutoff value for preoperative serum Cys-C levels was 0.95 mg/L, and patients were classified into low (≤ 0.95mg/L) and high Cys-C (> 0.95mg/L) groups, comprising 460 and 164 patients, respectively. The median follow-up period was 40 months (range: 1-101 months). During this period, 48 patients (7.7%) died, of which 30 (4.8%) died of cancer.

### Comparison of patient characteristics between low and high Cys‑C groups

Patients with high preoperative serum Cys-C levels were significantly in: older age, higher preoperative urea, CRE, and UA levels, and larger tumors. In addition, preoperative serum Cys-C levels were associated with gender, hypertension, cardiovascular disease, T stage, type of surgery, and surgical approach. However, there was no significant association between preoperative serum Cys-C levels and smoking, diabetes, tumor laterality, tumor subtype, N stage, Fuhrman grade, or BMI (*P* > 0.05) (**Table 1**).

### Prognostic value of the preoperative serum Cys‑C levels in RCC patients post-nephrectomy

During follow-up, 20 patients (4.3%) died in the low Cys-C group, of which 11 (2.4%) died of cancer. In contrast, 28 patients (17.1%) died in the high Cys-C group, of which 19 (11.6%) died of cancer.

**Figure 1:**
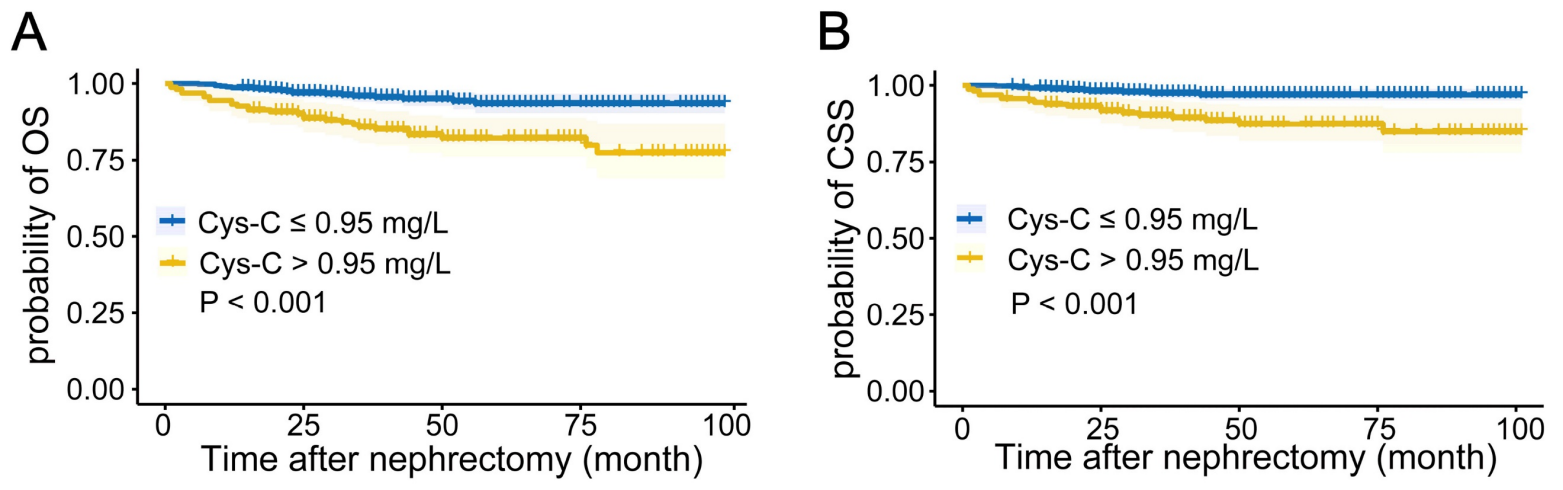
Kaplan-Meier curves for (A) overall survival, and (B) cancer-specific survival in patients with renal cell carcinoma post-nephrectomy stratified according to preoperative serum cystatin C levels.

The Kaplan-Meier curves depicted in **Figures 1A-B** indicated that patients with higher preoperative serum Cys-C levels tended to have a worse OS (*P* < 0.001) and CSS (*P* < 0.001).

**Figure 2:**
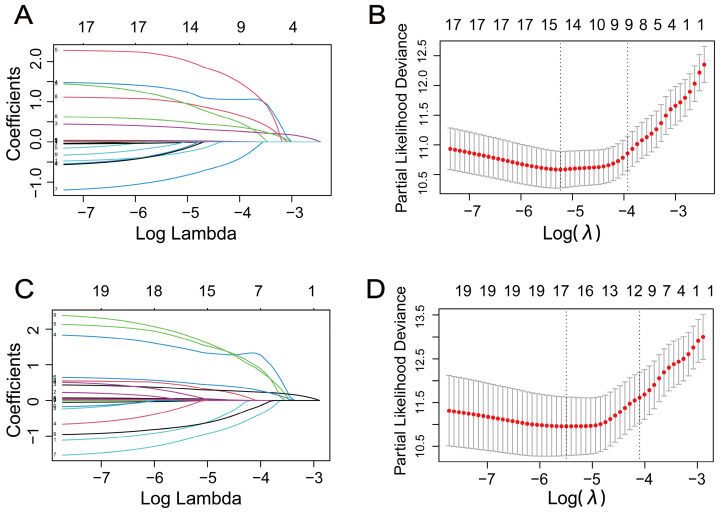
The selection process of factors affecting (A) (B) overall survival and (C) (D) cancer-specific survival by LASSO regression analysis. (A) (C) LASSO coefficient profiles of the 19 variables. (B) (D) the partial likelihood deviation curve versus log(λ).

To avoid overfitting and multicollinearity, we used LASSO regression combined with Cox survival analysis to identify independent predictors of OS and CSS. Regarding OS, LASSO regression identified 14 variables, which were included in Cox multivariate analysis (**Figures 2A-B**). According to **Table 2**, multivariate Cox regression analysis showed that age, smoking, diabetes, tumor size, tumor subtype, N stage, Fuhrman grade, and preoperative serum CRE and Cys-C levels were independent prognostic factors for OS. In **Table 3**, Cox proportional hazards regression models demonstrate the association between preoperative serum Cys-C levels and OS in patients with RCC. After adjusting for all covariates, the final multivariate model indicates that patients with high preoperative serum Cys-C levels had a multivariable HR of 2.254 (95% CI: 1.144, 4.439; *P* = 0.019), compared with those in the low Cys-C levels group.

Similarly, for CSS, 17 variables identified by LASSO regression were included in Cox multivariate analysis (**Figures 2C-D**). According to **Table 4**, the multivariate Cox regression analysis indicated that diabetes, hypertension, tumor size, tumor subtype, N stage, surgical approach, and preoperative UA and Cys-C were independent prognostic factors for CSS. In **Table 5**, Cox proportional hazards regression models demonstrate the association between preoperative serum Cys-C levels and CSS in patients with RCC. After adjusting for all covariates, the final multivariate model indicates that patients with high preoperative serum Cys-C levels had a multivariable HR of 3.621 (95% CI: 1.386, 9.456; *P* = 0.009), compared with those in the low Cys-C levels group.

### Predictive accuracy of established model

**Figure 3:**
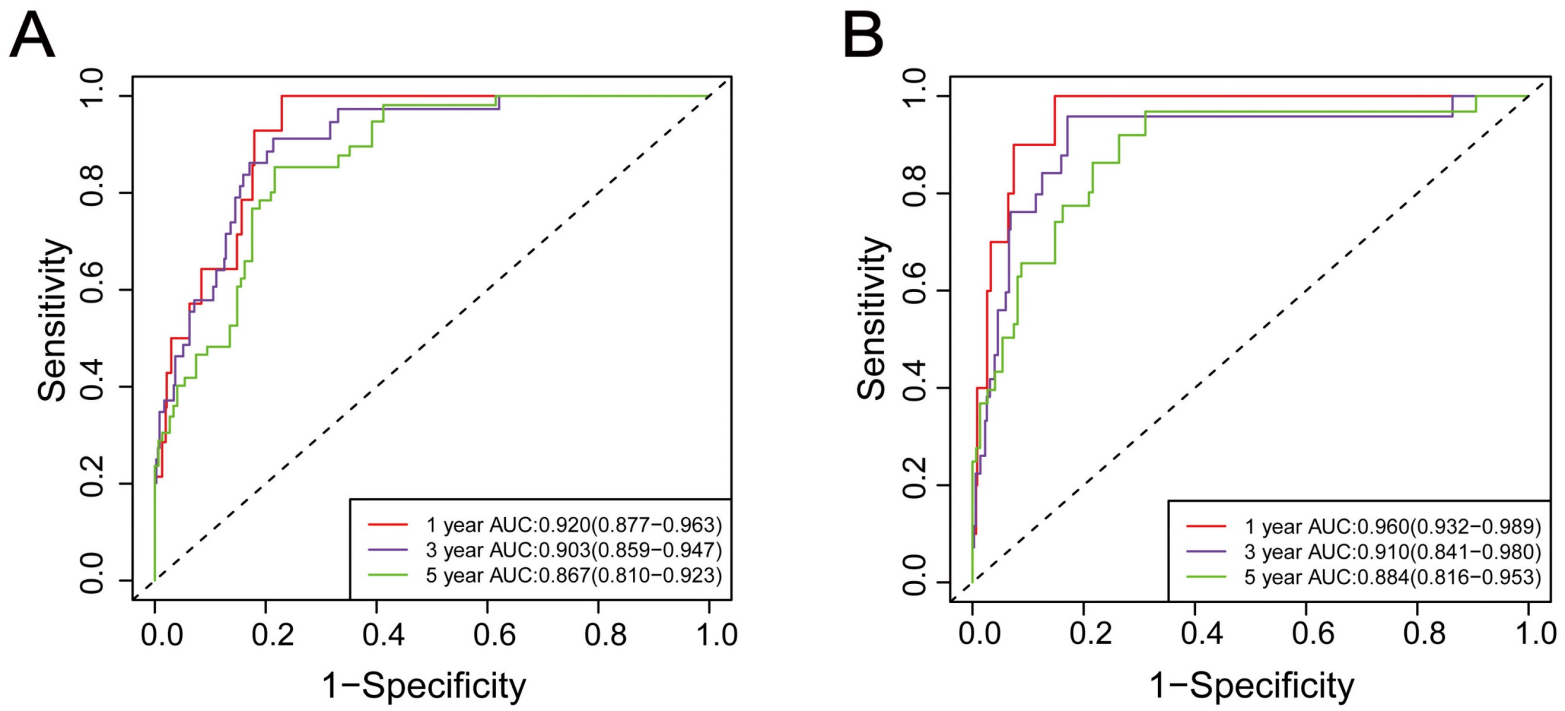
Time-dependent receiver operating characteristic curve analysis of the prognostic accuracy of Cox regression model for 1-year, 3-year, and 5-year overall survival (A) and 1-year, 3-year, and 5-year cancer-specific survival (B) in patients with renal cell carcinoma post-nephrectomy.

Time-dependent ROC curves and AUCs were used to evaluate the prediction accuracy of 1-year, 3-year, and 5-year OS and CSS in RCC patients undergoing nephrectomy. The AUCs of 1-year, 3-year, and 5-year OS were 0.920 (95%CI: 0.877-0.963), 0.903 (95%CI: 0.859-0.947), and 0.867 (95%CI: 0.810-0.923), as shown in **Figure 3A**. The AUCs of 1-year, 3-year, and 5-year CSS were 0.960 (95%CI: 0.932-0.989), 0.910 (95%CI: 0.841-0.980), and 0.884 (95%CI: 0.816-0.953), as shown in **Figure 3B**.

These results demonstrated that the Cox regression model, including preoperative serum Cys-C, performed well in predicting accuracy of survival in RCC patients.

## Discussion

In our study, we used 0.95 mg/L as preoperative serum Cys-C optimal cutoff concentration and demonstrated that elevated serum Cys-C level was associated with worse OS and CSS among RCC patients. High Cys-C level was identified as an unfavorable prognostic indicator, independent of other clinicopathological features of RCC.

There is increasing interest in the role of Cys-C in renal cancer. Guo *et al.* 21 enrolled 325 RCC patients who underwent nephrectomy and retrospectively evaluated the association between preoperative serum Cys-C levels and clinicopathological parameters and survival. They discovered that high serum Cys-C level was related to Fuhrman grade, TNM stage, and pathological types, and it was an independent prognostic factor for OS and disease-free survival. Similarly, Bodnar *et al.* 22 evaluated the influence of serum Cys-C on the efficacy of everolimus in patients with metastatic RCC and concluded that patients with high pre-treatment serum Cys-C had worse OS than those with low serum Cys-C. These findings are consistent with our results, suggesting that measurement of preoperative serum Cys-C might be a straightforward method to determine the prognosis of RCC patients. Consequently, patients with elevated preoperative serum Cys-C levels should be closely followed up. Moreover, Cys-C has been shown to be expressed in RCC tissues. Researchers used immunohistochemistry and Western blot assays to evaluate Cys-C expression levels in 253 clear cell RCC (ccRCC) tissues 23. Their results indicated that high Cys-C expression level in ccRCC tissues might be an adverse prognostic indicator.

Despite the well-documented association between preoperative serum Cys-C levels and cancers prognosis, the potential mechanisms remain unclear. Previous studies have revealed the complex effects of Cys-C on tumor cell growth and dissemination. Huh *et al.* 24 first highlighted the promotion effects of Cys-C on tumor growth. Subsequent studies demonstrated that lysosomal cysteine proteinases appear to participate in various immunoreaction processes, including the maturation of antigen-presenting cells, antigen processing, and presentation to T cells 25, 26. Their inhibition might enable tumor cells to escape immune surveillance 14, 27. Therefore, Cys-C, as a potent inhibitor of lysosomal cysteine proteinases, may indirectly promote cancer cells growth and spread 28.

Strengths of this study include its large sample size and it is the first to evaluate the association between preoperative serum Cys-C levels and CSS in RCC patients. However, there are several limitations must be admitted. Firstly, our study is a single-center study of Chinese RCC patients. Secondly, it is impossible for us to rule out the influence of some potential confounding factors. Thirdly, the follow-up period was relatively short, and only 48 patients (7.7%) died by the end of the investigation. Therefore, our results need to be validated in multicenter studies with longer follow-up periods.

## Conclusions

High preoperative serum Cys-C level is an independent adverse prognostic factor of OS and CSS for RCC patients who underwent partial or radical nephrectomy. This finding can assist urologists in better stratifying patients and guiding their personalized therapy. Preoperative serum Cys-C could be an effective indicator to evaluate the prognosis of RCC patients undergoing nephrectomy due to its noninvasiveness and reproducibility.

## Figures and Tables

**Table 1:** Comparison of baseline characteristics of RCC patients between the low and high Cys-C groups

Variables	All patients (n=624)	Cys-C ≤ 0.95mg/L (n=460)	Cys-C > 0.95mg/L (n=164)	*P*
	n (%)			
Gender				0.001*
Male	408(65.4)	284(61.7)	124(75.6)	
Female	216(34.6)	176(38.3)	40(24.4)	
Smoking history				0.523
Yes	167(26.8)	120(26.1)	47(28.7)	
No	457(73.2)	340(73.9)	117(71.3)	
Diabetes				0.756
Yes	98(15.7)	71(15.4)	27(16.5)	
No	526(84.3)	389(84.6)	137(83.5)	
Hypertension				0.007*
Yes	253(40.5)	172(37.4)	81(49.4)	
No	371(59.5)	288(62.6)	83(50.6)	
Cardiovascular disease				<0.001*
Yes	57(9.1)	30(6.5)	27(16.5)	
No	567(90.9)	430(93.5)	137(83.5)	
Laterality				0.789
Left	295(47.3)	216(47.0)	79(48.2)	
Right	329(52.7)	244(53.0)	85(51.8)	
Tumor subtype				0.066
Clear	576(92.3)	430(93.5)	146(89.0)	
Non-clear	48(7.7)	30(6.5)	18(11.0)	
T stage				0.001*
T1	544(87.2)	414(90.0)	130(79.3)	
T2	58(9.3)	36(7.8)	22(13.4)	
T3-T4	22(3.5)	10(2.2)	12(7.3)	
N stage				0.656
N0/Nx	618(99.0)	456(99.1)	162(98.8)	
N+	6(1.0)	4(0.9)	2(1.2)	
Fuhrman grade				0.618
1	114(18.3)	84(18.3)	30(18.3)	
2	367(58.8)	276(60.0)	91(55.5)	
3	123(19.7)	85(18.5)	38(23.2)	
4	20(3.2)	15(3.2)	5(3.0)	
Type of surgery				0.007*
RN	419(67.1)	295(64.1)	124(75.6)	
PN	205(32.9)	165(35.9)	40(24.4)	
Surgical approach				0.002*
Open	70(11.2)	41(8.9)	29(17.7)	
Laparoscope	554(88.8)	419(91.1)	135(82.3)	
	Mean ± SD			
Urea (mmol/L)	5.09 ± 1.42	4.91 ± 1.22	5.61 ± 1.76	<0.001*
CRE (μmol/L)	71.44 ± 17.11	67.58 ± 13.44	82.26 ± 21.21	<0.001*
UA (μmol/L)	328.64 ± 92.05	320.82 ± 89.58	350.58 ± 95.56	<0.001*
Age (years)	57.49 ± 10.71	56.01 ± 10.72	61.63 ± 9.56	<0.001*
BMI (kg/m^2^)	25.02 ± 3.41	25.12 ± 3.49	24.71 ± 3.15	0.180
Tumor size(cm)	4.43 ± 2.35	4.24 ± 2.22	4.97 ± 2.61	0.002*

Continuous variables were analyzed by the Student's t-test, while categorical variables were analyzed by the Chi-squared test. Abbreviations: Cys-C: cystatin C; RN: radical nephrectomy; PN: partial nephrectomy; SD: standard deviation; BMI: body mass index; CRE: creatinine; UA: uric acid * indicates *P*<0.05

**Table 2:** Multivariable Cox regression analysis of OS in RCC patients post-nephrectomy

		95%CI for HR		
Variables	HR	Lower	Upper	*P*
Age (years)	1.047	1.012	1.083	0.009*
BMI (kg/m^2^)	0.963	0.861	1.078	0.514
CRE(μmol/L)	1.020	1.005	1.035	0.010*
Smoking history (no vs yes)	3.495	1.874	6.519	<0.001*
Diabetes (no vs yes)	4.136	1.887	9.063	<0.001*
Hypertension (no vs yes)	0.592	0.307	1.144	0.119
Cardiovascular disease (no vs yes)	0.652	0.243	1.754	0.397
Tumor size (cm)	1.553	1.312	1.839	<0.001*
Tumor subtype (non-clear vs clear)	0.278	0.124	0.622	0.002*
T stage				0.196
T1	Reference			
T2	0.506	0.157	1.632	0.254
T3-T4	0.336	0.102	1.101	0.072
N stage (N0/Nx vs N+)	11.602	3.103	43.383	<0.001*
Fuhrman grade				0.012*
1	Reference			
2	1.546	0.494	4.841	0.454
3	2.785	0.904	8.578	0.074
4	7.383	1.831	29.771	0.005*
Surgical approach (Open vs Laparoscope)	0.645	0.304	1.366	0.252
Cys-C (≤0.95 vs >0.95)	2.259	1.170	4.360	0.015*

Abbreviations: OS: overall survival; HR: hazard ratio; CI: confidence interval; BMI: body mass index; CRE: creatinine; Cys-C: cystatin C * indicates *P*<0.05

**Table 3:** Association between preoperative serum Cys-C levels and OS in patients with renal cell carcinoma

Cys-C	Non-adjusted	*P*	Model Ⅰ	*P*	Model Ⅱ	*P*	Model Ⅲ	*P*
Cys-C ≤ 0.95	1 (reference)		1 (reference)		1 (reference)		1 (reference)	
Cys-C > 0.95	3.564 (2.003, 6.343) ^b^	**<0.001^a^**	2.842 (1.559, 5.182)	**0.001**	3.525 (1.913, 6.495)	**<0.001**	2.254 (1.144, 4.439)	**0.019**

^a^ Obtained by using multivariable Cox regression model ^b^ Hazard ratios (95% confidence interval) (all such values) Non-adjusted model adjusted for: None. Model Ⅰ was adjusted for age, gender and BMI. Model Ⅱ was adjusted for age, gender, BMI, laterality, smoking history, diabetes, hypertension, and cardiovascular disease. Model Ⅲ was adjusted for age, gender, BMI, laterality, smoking history, diabetes, hypertension, cardiovascular disease, urea, CRE, UA, tumor size, tumor subtype, T stage, N stage, Fuhrman grade, type of surgery, and surgical approach. Abbreviations: OS: overall survival; Cys-C: cystatin C; BMI: body mass index; CRE: creatinine; UA: uric acid

**Table 4:** Multivariable Cox regression analysis of CSS in RCC patients post-nephrectomy

		95%CI for HR		
Variables	HR	Lower	Upper	*P*
Age (years)	1.042	0.995	1.091	0.077
BMI (kg/m^2^)	0.979	0.856	1.120	0.760
Urea (mmol/L)	1.099	0.826	1.463	0.516
CRE (μmol/L)	1.023	0.997	1.049	0.082
UA (μmol/L)	0.993	0.988	0.999	0.024*
Smoking history (no vs yes)	2.146	0.940	4.900	0.070
Diabetes (no vs yes)	13.366	4.683	38.148	<0.001*
Hypertension (no vs yes)	0.291	0.120	0.705	0.006*
Laterality (left vs right)	1.225	0.528	2.839	0.637
Tumor size (cm)	1.501	1.193	1.889	0.001*
Tumor subtype (non-clear vs clear)	0.201	0.070	0.580	0.003*
T stage				0.433
T1	Reference			
T2	0.727	0.140	3.778	0.704
T3-T4	0.359	0.071	1.806	0.214
N stage (N0/Nx vs N+)	8.528	1.383	52.598	0.021*
Fuhrman grade				0.058
1	Reference			
2	6.807	0.776	59.705	0.083
3	7.455	0.866	64.189	0.067
4	26.506	2.263	310.462	0.009*
Type of surgery (PN vs RN)	2.043	0.410	10.181	0.383
Surgical approach (Open vs Laparoscope)	0.314	0.128	0.769	0.011*
Cys-C (≤0.95vs>0.95)	3.617	1.419	9.222	0.007*

Abbreviations: CSS: cancer-specific survival; HR: hazard ratio; CI: confidence interval; BMI: body mass index; CRE: creatinine; UA: uric acid; PN: partial nephrectomy; RN: radical nephrectomy; Cys-C: cystatin C * indicates *P*<0.05

**Table 5:** Association between preoperative serum Cys-C levels and CSS in patients with renal cell carcinoma

Cys-C	Non-adjusted	*P*	Model Ⅰ	*P*	Model Ⅱ	*P*	Model Ⅲ	*P*
Cys-C ≤ 0.95	1 (reference)		1 (reference)		1 (reference)		1 (reference)	
Cys-C > 0.95	4.506 (2.139, 9.492) ^b^	**<0.001^a^**	3.893 (1.785, 8.489)	**0.001**	5.283 (2.388, 11.685)	**<0.001**	3.621 (1.386, 9.456)	**0.009**

^a^ Obtained by using multivariable Cox regression model ^b^ Hazard ratios (95% confidence interval) (all such values) Non-adjusted model adjusted for: None. Model Ⅰ was adjusted for age, gender, and BMI. Model Ⅱ was adjusted for age, gender, BMI, laterality, smoking history, diabetes, hypertension, and cardiovascular disease. Model Ⅲ was adjusted for age, gender, BMI, laterality, smoking history, diabetes, hypertension, cardiovascular disease, urea, CRE, UA, tumor size, tumor subtype, T stage, N stage, Fuhrman grade, type of surgery, and surgical approach. Abbreviations: CSS: cancer-specific survival; Cys-C: cystatin C; BMI: body mass index; CRE: creatinine; UA: uric acid

## Abbreviations

Cys-C: cystatin C
RCC: renal cell carcinoma
BMI: body mass index
CRE: creatinine
UA: uric acid
OS: overall survival
CSS: cancer-specific survival
ROC: receiver operating characteristic
AUC: area under the curve
HR: hazard ratio
CI: confidence interval
SD: standard deviation
RN: radical nephrectomy
PN: partial nephrectomy
ccRCC: clear cell renal cell carcinoma
